# Effects of Stocking Density on Growth, Hepatic Antioxidant and Inflammatory Responses, and Intestinal Barrier Function in Cage-Cultured Large Yellow Croaker (*Larimichthys crocea*)

**DOI:** 10.3390/antiox15101257

**Published:** 2026-10-01

**Authors:** Ruoyu Chai, Zheng Liu, Huihui Zhou, Chengdong Liu, Kangsen Mai, Gen He, Xuan Wang

**Affiliations:** 1 Key Laboratory of Mariculture, Ministry of Education, Ocean University of China, Qingdao 266003, China; 2 Key Laboratory of Aquaculture Nutrition, Ministry of Agriculture and Rural Affairs, Ocean University of China, Qingdao 266003, China

**Keywords:** *Larimichthys crocea*, stocking density, chronic stress, Nrf2, NF-κB signaling, tight junction protein, intestinal barrier

## Abstract

This study investigated the effects of stocking density on growth, antioxidant responses, hepatic inflammatory signaling, and intestinal barrier function in cage-cultured large yellow croaker (*Larimichthys crocea*). Fish (~288 g) were reared for 8 weeks at low (5.73 kg/m^3^), medium (8.64 kg/m^3^), or high (11.55 kg/m^3^) initial stocking densities. High density reduced growth and survival and increased feed conversion ratio, serum cortisol, glucose, and malondialdehyde, while decreasing total antioxidant capacity and superoxide dismutase activity (*p* < 0.05). Hepatic Nrf2, SOD, CAT, and GPX expression decreased, whereas Keap1 expression increased at high density. High density also increased pro-inflammatory cytokine expression and NF-κB p65 phosphorylation, decreased anti-inflammatory factors and IκB abundance, shortened intestinal villi, reduced ZO-1, Occludin, and Claudin-1 abundance, and increased serum diamine oxidase, D-lactate, and endotoxin (*p* < 0.05). Medium-density fish showed generally comparable responses to low-density fish for most endpoints. These findings indicate that high stocking density induces physiological stress and oxidative imbalance associated with an impaired hepatic Nrf2-related antioxidant response, enhanced NF-κB inflammatory signaling, and impaired intestinal barrier integrity in large yellow croaker.

## 1. Introduction

Aquaculture has become the fastest-growing food production sector and now supplies more than half of the fish consumed worldwide, with intensification being the dominant strategy to increase yield per unit water volume [1,2]. Stocking density is one of the most critical management factors in intensive aquaculture, because it simultaneously determines production efficiency, fish welfare and economic returns [3,4]. However, when the stocking density exceeds the optimal range of a given species and culture system, crowding acts as a chronic stressor, leading to growth retardation, impaired feed utilization, immunosuppression and increased disease susceptibility [5,6,7]. Such density-dependent detrimental effects have been documented in a wide range of farmed species, including turbot (*Scophthalmus maximus*) [8], Chinese sturgeon (*Acipenser sinensis*) [9], grass carp (*Ctenopharyngodon idella*) [10], Nile tilapia (*Oreochromis niloticus*) [11], channel catfish (*Ictalurus punctatus*) [12] and gilthead sea bream (*Sparus aurata*) [13]. Therefore, determining the optimal stocking density and elucidating the physiological mechanisms underlying crowding-induced damage are essential for the sustainable development of intensive fish farming.

The adverse effects of high stocking density are initiated by the activation of the neuroendocrine stress response [14]. In fish, crowding stress activates the hypothalamic–pituitary–interrenal (HPI) axis, resulting in elevated circulating cortisol, which in turn mobilizes energy reserves and induces secondary responses such as hyperglycemia [15,16,17]. Persistent activation of the stress axis can also disturb the balance between the production and scavenging of reactive oxygen species (ROS), leading to oxidative stress and subsequent damage to lipids, proteins, and DNA [18,19]. The Keap1/Nrf2 system is a major redox-sensitive regulatory mechanism involved in cellular defense against oxidative stress. Under basal conditions, Kelch-like ECH-associated protein 1 (Keap1) negatively regulates nuclear factor erythroid 2-related factor 2 (Nrf2) by promoting its ubiquitination and proteasomal degradation. In response to oxidative stimuli, Nrf2 can escape Keap1-mediated degradation, accumulate, and translocate into the nucleus, where it regulates the transcription of antioxidant and cytoprotective genes [20,21]. In aquatic organisms, the Nrf2/Keap1 system is also an important component of the antioxidant response, and its downstream antioxidant defense involves enzymes such as superoxide dismutase (SOD), catalase (CAT), and glutathione peroxidase (GPX) [22,23]. Therefore, alterations in the Keap1/Nrf2 system and antioxidant enzyme expression may provide a molecular basis for the oxidative imbalance induced by chronic crowding stress. Importantly, chronic stress and oxidative damage are tightly coupled with immune dysfunction [24]. The inflammatory homeostasis of fish is orchestrated by pro-inflammatory cytokines, such as tumor necrosis factor-α (TNF-α), interleukin-1β (IL-1β) and IL-6, and anti-inflammatory factors, including IL-10, transforming growth factor-β (TGF-β) and arginase 1 [25]. The transcription of pro-inflammatory cytokines is predominantly controlled by the nuclear factor-κB (NF-κB) signaling pathway: Under basal conditions, NF-κB is sequestered in the cytoplasm by its inhibitor IκB. Upon stimulation, IκB is phosphorylated and degraded, allowing NF-κB nuclear translocation. The released NF-κB p65 is further activated through phosphorylation (in the cytoplasm or nucleus), which promotes its interaction with coactivators and initiates inflammatory gene transcription [26]. Long-term crowding stress has been reported to up-regulate pro-inflammatory cytokines and down-regulate anti-inflammatory cytokines in grass carp [10]; however, whether the NF-κB pathway mediates density-induced inflammation remains poorly characterized in marine-farmed fish.

Beyond systemic stress and immune responses, the intestine has increasingly been recognized as a primary target organ of environmental and husbandry stressors [27]. The fish intestine is not only the major site of nutrient digestion and absorption, but also the largest immune organ and a critical physical barrier separating the host from luminal antigens, toxins and pathogens [28,29,30]. The absorptive capacity of the intestine depends largely on its morphological integrity, commonly evaluated by villus height and crypt depth, whereas the physical barrier is maintained at the molecular level by tight junction protein complexes: the transmembrane proteins occludin and claudins seal the paracellular space between epithelial cells, while the intracellular scaffold protein zonula occludens-1 (ZO-1) links these proteins to the actin cytoskeleton [31,32]. Disruption of tight junction proteins increases intestinal permeability, which can be monitored by plasma markers such as D-lactate and endotoxin. Elevated circulating endotoxin, in turn, is a potent activator of pattern-recognition receptor (TLR4) signaling and can trigger systemic and hepatic inflammation, establishing a “gut–liver axis” through which intestinal barrier dysfunction and systemic inflammation mutually reinforce each other [33]. Although stocking density has been shown to affect intestinal morphology and microbiota in several fish species [13], the effects of crowding stress on intestinal tight junction proteins and their relationship with systemic inflammatory signaling remain largely unknown.

The large yellow croaker (*Larimichthys crocea*), a member of the family Sciaenidae, is endemic to the coastal waters of East Asia and is currently the most productive marine-farmed fish species in China, with an aquaculture production of approximately 257,683 tons in 2023 [34]. Driven by limited coastal space and increasing market demand, farmers tend to maximize stocking density, and density-related problems such as reduced growth, chronic stress and frequent disease outbreaks have become major constraints on the sustainable development of the large yellow croaker industry. Previous studies have revealed that high stocking density impairs growth performance, digestive and metabolic enzyme activities [35], muscle nutritional quality and serum immune responses [36] of large yellow croaker, and transcriptome analysis has identified immune-related genes responsive to acute high-density stress in this species [37]. However, the integrated effects of stocking density on physiological stress, antioxidant capacity, hepatic inflammatory signaling, and intestinal barrier function remain poorly understood in large yellow croaker, particularly with respect to NF-κB signaling and intestinal tight-junction proteins.

Based on the evidence summarized above, we hypothesized that excessive stocking density acts as a chronic stressor that disrupts redox homeostasis and the hepatic Nrf2-related antioxidant response, promotes NF-κB-associated inflammatory signaling, and impairs intestinal barrier integrity in large yellow croaker. To test this hypothesis, the present study investigated growth performance, physiological stress and systemic antioxidant status, as well as hepatic expression of Nrf2-related antioxidant genes, inflammatory signaling and intestinal barrier function in cage-cultured large yellow croaker exposed to different stocking densities.

## 2. Materials and Methods

All procedures of animal rearing and handling were conducted in accordance with the guidelines of the Institutional Animal Care and Use Committee of Ocean University of China.

### 2.1. Experimental Fish and Rearing Management

Large yellow croaker were obtained from a commercial fish farm in Ningde, Fujian Province, China, and acclimated for two weeks before the trial. After acclimation, a total of 1215 healthy fish of similar size (initial body weight 287.63 ± 1.21 g) were randomly distributed into nine net cages (effective water volume 4.5 m^3^ each). Three stocking density treatments were established, a low-density group (LD, 90 fish per cage, initial density of 5.73 kg/m^3^), a medium-density group (MD, 135 fish per cage, 8.64 kg/m^3^) and a high-density group (HD, 180 fish per cage, 11.55 kg/m^3^), with three replicate cages per treatment. The three stocking densities were selected based on previously reported density ranges for large yellow croaker [36], with minor adjustments according to the fish size and cage conditions used in the present study. The feeding trial lasted for 8 weeks. During the trial, fish were fed twice daily (05:00 and 17:00) to apparent satiation with a commercial diet for large yellow croaker (Ao Hua, Zhangzhou, Fujian, China). The complete ingredient formulation was proprietary and was not disclosed by the manufacturer; therefore, only the available proximate composition and key nutritional specifications are reported (Table 1). Uneaten feed was collected, dried and weighed to calculate the feed intake. Water temperature, dissolved oxygen, salinity and pH were measured in situ in each cage (at approximately 0.5 m depth) (twice daily at 05:00 and 17:00, before feeding) throughout the 8-week trial using a portable multiparameter water quality meter (Pro 20i, YSI, Yellow Springs, OH, USA). During the experimental period, the water temperature was 23.4 ± 0.2 °C, salinity was 21.5 ± 0.3‰, dissolved oxygen was 7.2 ± 0.1 mg/L, pH was 8.0 ± 0.1, and the total ammonia nitrogen was 0.03 ± 0.01 mg/L. At the end of the feeding trial, fish were fasted for 24 h and then counted and batch-weighed in each cage. Growth performance and feed utilization were calculated as follows:

Weight gain rate (WGR, %) = (final body weight − initial body weight)/initial body weight × 100;

Specific growth rate (SGR, %/day) = (ln final body weight − ln initial body weight)/experimental days × 100;

Feeding rate (FR, % body weight/day) = dry feed intake/[(initial body weight + final body weight)/2 × experimental days] × 100;

Feed conversion ratio (FCR) = dry feed intake/(final body weight − initial body weight);

Survival rate (SR, %) = final fish number/initial fish number × 100.

### 2.2. Sample Collection

At the end of the trial, after a 24-h fasting period, two fish were randomly sampled from each replicate cage, resulting in six fish per treatment (*n* = 6) and anesthetized with MS-222 (80 mg/L) [38,39]. To minimize the influence of handling stress on serum cortisol and glucose, all samplings were performed between 07:00 and 11:00 after the 24-h fasting. Fish from each cage were captured with a single rapid net haul, and blood was collected from the caudal vein within 1 min of capture for each individual fish. The sampling order of the nine cages was randomized, and the same trained personnel performed capture, anesthesia and blood collection throughout the sampling. Blood was collected from the caudal vein using disposable syringes, kept at 4 °C for 4 h, and then centrifuged at 3000 *g* for 10 min at 4 °C to obtain serum, which was stored at −80 °C until analysis. After blood collection, fish were dissected on ice. Liver and gut samples were rapidly collected, frozen in liquid nitrogen and stored at −80 °C for gene expression and Western blot analyses. Mid-intestine segments (approximately 1 cm) were excised and fixed in 4% paraformaldehyde for histological observation.

### 2.3. Serum Biochemical, Antioxidant and Intestinal Permeability Indices

Serum cortisol was determined with a fish cortisol ELISA kit (Cat. No. H094-1-1, Nanjing Jiancheng Bioengineering Institute, Nanjing, China) according to the manufacturer’s instructions; absorbance was read at 450 nm with a microplate reader. Serum glucose was measured by the glucose oxidase method (Cat. No. A154-1-1). T-AOC (Cat. No. A015-1-1), SOD (Cat. No. A001-3-1) and MDA (Cat. No. A003-1-2) were determined with commercial kits (Nanjing Jiancheng, China) following the manufacturer’s protocols; SOD activity was assayed by the [WST-1/xanthine oxidase] method and MDA by the thiobarbituric acid method. Serum DAO (Cat. No. A088-2-1), D-lactate (Cat. No. A019-3-1) and endotoxin (Cat. No. E039-1-1) were measured with ELISA kits. All assays were performed in triplicate.

### 2.4. Quantitative Real-Time PCR

The hepatic mRNA expression of antioxidant- and inflammation-related genes was analyzed using quantitative real-time PCR. Total RNA was extracted from liver samples using TRIzol™ reagent (Invitrogen, Carlsbad, CA, USA). RNA integrity was confirmed by 1.2% denaturing agarose gel electrophoresis, and RNA quantity was measured by spectrophotometry. The cDNA was synthesized using the PrimeScript™ RT reagent kit (Takara, Gunma, Japan). Quantitative real-time PCR was performed with SYBR Green Premix (Takara, Japan) on a CFX96 Touch Real-Time PCR Detection System (Bio-Rad Laboratories, Hercules, CA, USA). The amplification program consisted of 95 °C for 30 s, followed by 40 cycles of 95 °C for 5 s and 60 °C for 30 s, followed by melting-curve analysis to confirm amplification specificity. Each sample was analyzed in technical triplicate. Amplification efficiencies of all primer pairs ranged from 95% to 105%. The stability of β-actin was verified across treatments, with no significant difference in Ct values (*p* > 0.05). Primer sequences are listed in Table 2. Relative gene expression was calculated using the 2^−ΔΔCt^ method, with the LD group normalized to 1.0.

### 2.5. Western Blot Analysis

Western blot was used to determine the activation of the hepatic NF-κB pathway and the abundance of intestinal tight junction proteins. Briefly, tissues were homogenized with RIPA buffer containing protease and phosphatase inhibitors, and the lysate was incubated on a shaker for 30 min at 4 °C. Then the lysate was centrifuged at 12,000 *g* for 10 min at 4 °C, and the supernatant was collected. The protein concentration was measured with a BCA protein detection kit (Beyotime, Shanghai, China). For Western blot, 30 μg of protein per lane was separated on 10% SDS-PAGE gels and transferred to PVDF membranes (0.45 μm, MilliporeSigma, Burlington, MA, USA). The membranes were blocked and incubated with primary antibodies against phospho-NF-κB p65 (Ser536) (Cell Signaling Technology, Cat: 3033T), NF-κB p65 (Cell Signaling Technology, Cat: 8242T), IκB (Cell Signaling Technology, Cat: 4814T), ZO-1 (Cell Signaling Technology, Cat: 5406S), Occludin (Cell Signaling Technology, Cat: 91131T), Claudin-1 (Cell Signaling Technology, Cat:4933T) and GAPDH (Cell Signaling Technology, Cat: 2118T), followed by incubation with horseradish peroxidase-conjugated secondary antibodies. Protein signals were captured using an enhanced chemiluminescence reagent (Biosharp Biotechnology, Wuhan, China) and exposed to X-ray film. Band densities were analyzed using NIH ImageJ software (version 1.63). The ratio of p-NF-κB (Ser536) to total NF-κB and the abundance of IκB, ZO-1, Occludin and Claudin-1 relative to GAPDH were calculated and normalized to the LD group (set as 1.0).

### 2.6. Intestinal Morphology

The mid-intestine segments fixed in 4% paraformaldehyde were dehydrated in a graded ethanol series, embedded in paraffin, and sectioned at 5 μm thickness. The sections were stained with hematoxylin and eosin (H&E) and observed under a light microscope (Olympus BX51, Tokyo, Japan). We measured villus height using Image-Pro Plus 6.0 software (Media Cybernetics, Rockville, MD, USA). For each fish, we selected at least five fields of view, and we measured six fish per treatment.

### 2.7. Statistical Analysis

The experimental unit and number of biological replicates differed among measurements. Growth performance and feed utilization were analyzed using the cage as the experimental unit (*n* = 3 replicate cages per treatment). Serum biochemical, antioxidant and intestinal permeability indices, hepatic gene expression, and intestinal morphology were analyzed using individual fish as biological replicates (*n* = 6 fish per treatment, with two fish sampled from each replicate cage). Western blot analyses were performed with three biological replicates per treatment (*n* = 3).

The results were expressed as mean ± standard error (SE). All data were assessed for normality and homoscedasticity before analysis. Subsequently, a one-way analysis of variance (ANOVA) was conducted on the data obtained from the experimental group. If the one-way ANOVA results revealed significant differences (*p* < 0.05), Tukey’s test was employed for additional pairwise comparisons of the data. All data processing was carried out using SPSS 26.0 (IBM, Armonk, NY, USA) statistical software. Values with different superscripts are significantly different (*p* < 0.05).

## 3. Results

### 3.1. Effects of Stocking Density on Growth Performance and Feed Utilization of Large Yellow Croaker

The growth performance and feed utilization of large yellow croaker reared at different stocking densities are shown in Table 3. No significant difference was observed in the initial body weight among the three groups (*p* > 0.05). After the 8-week feeding trial, the final body weight of the MD group was significantly higher than that of the LD group, and both were significantly higher than that of the HD group (*p* < 0.05). The weight gain rate and specific growth rate of the LD and MD groups were comparable (*p* > 0.05), and both were significantly higher than those of the HD group (*p* < 0.05). The feeding rate was significantly lower in the HD group than in the LD and MD groups (*p* < 0.05). The feed conversion ratio of the HD group was significantly higher than those of the LD and MD groups (*p* < 0.05). The survival rate of the HD group was significantly lower than those of the LD and MD groups (*p* < 0.05).

### 3.2. Effects of Stocking Density on Serum Stress Indicators

**Figure 1:**
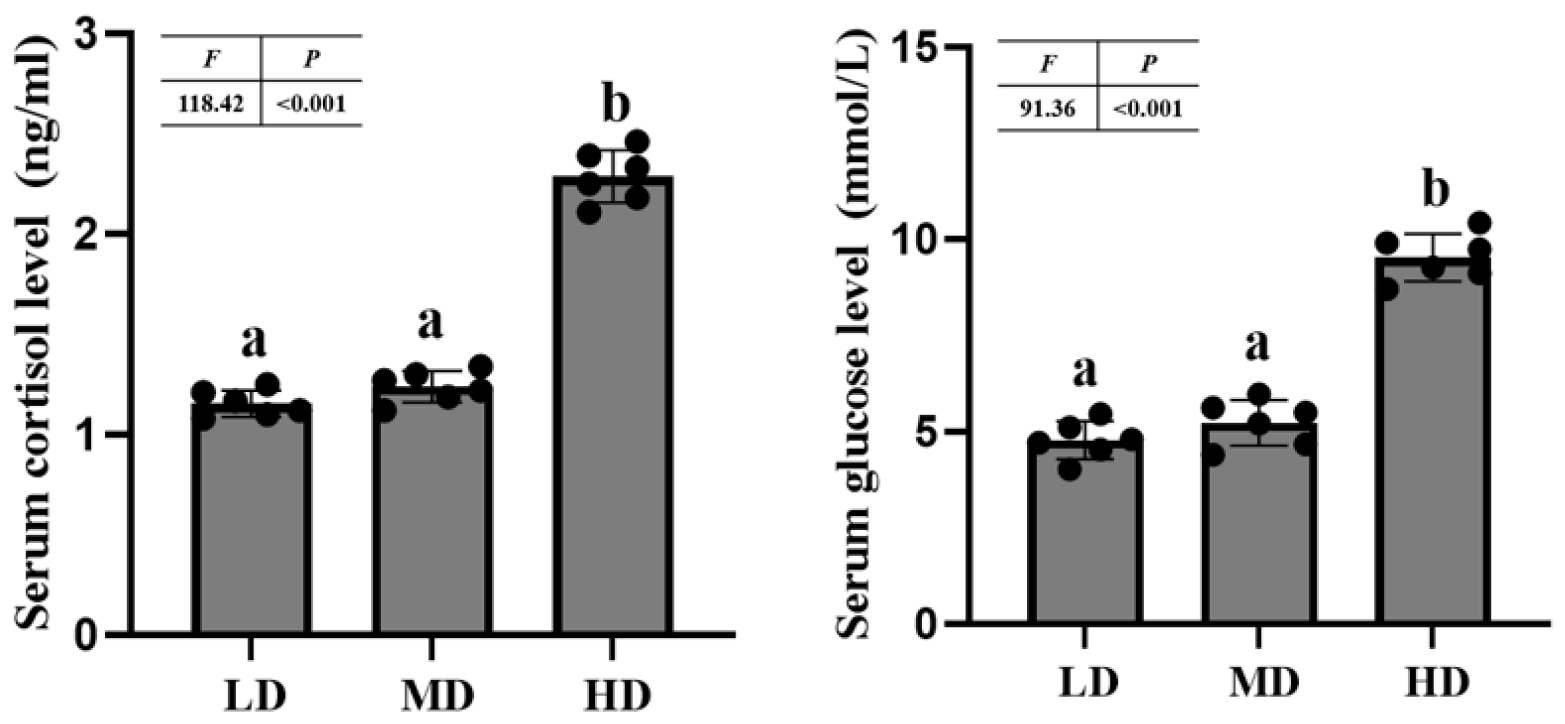
Effects of stocking density on serum stress indicators of large yellow croaker. Serum cortisol and glucose levels were measured after the 8-week feeding trial. Values are presented as mean ± SE (*n* = 6). Groups labeled with different letters are significantly different (*p* < 0.05). Dots represent individual biological replicates.

As shown in Figure 1, the serum cortisol level of the HD group was significantly higher than those of the LD and MD groups (*p* < 0.05), while no significant difference was detected between the LD and MD groups (*p* > 0.05). Similarly, the serum glucose content was significantly elevated in the HD group compared with the LD and MD groups (*p* < 0.05).

### 3.3. Effects of Stocking Density on Serum Antioxidant Capacity

**Figure 2:**
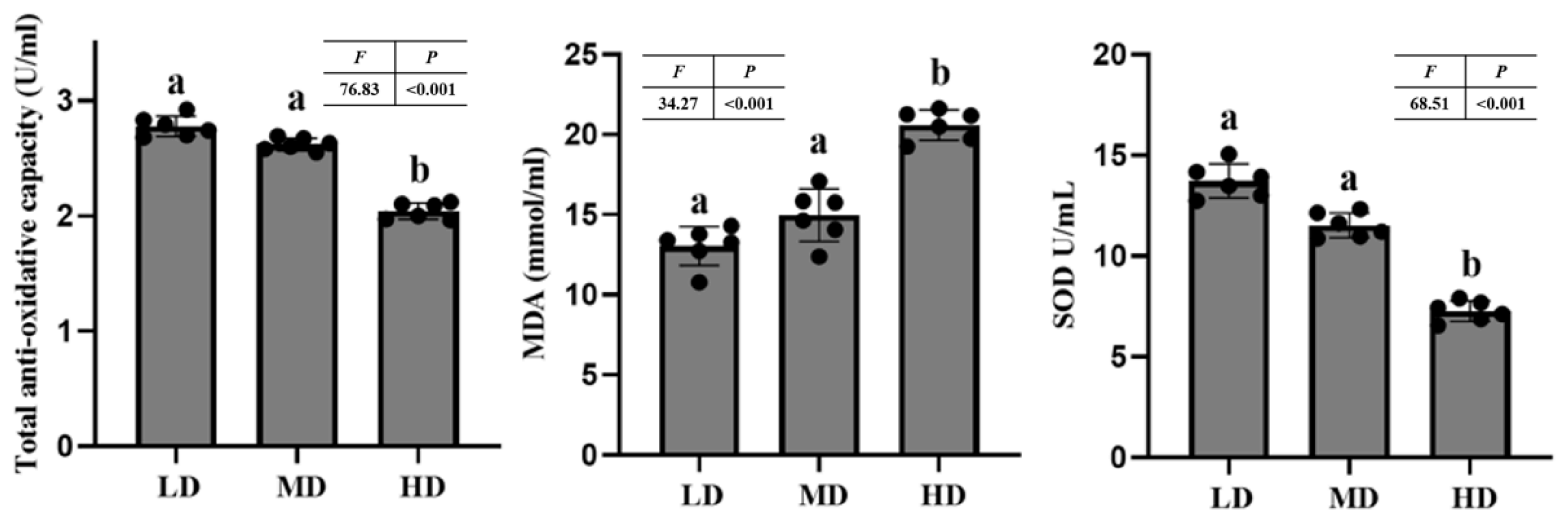
Effects of stocking density on serum antioxidant capacity of large yellow croaker. Total antioxidant capacity (T-AOC), superoxide dismutase (SOD) activity and malondialdehyde (MDA) content in serum were measured. Values are presented as mean ± SE (*n* = 6). Groups labeled with different letters are significantly different (*p* < 0.05). Dots represent individual biological replicates.

The serum antioxidant indices of large yellow croaker were markedly affected by stocking density (Figure 2). The total antioxidant capacity (T-AOC) and superoxide dismutase (SOD) activity did not differ between the LD and MD groups (*p* > 0.05), but were significantly decreased in the HD group (*p* < 0.05). Conversely, the serum malondialdehyde (MDA) content was significantly higher in the HD group than in the LD and MD groups (*p* < 0.05).

### 3.4. Effects of Stocking Density on Hepatic Expression of Antioxidant-Related Genes

**Figure 3:**
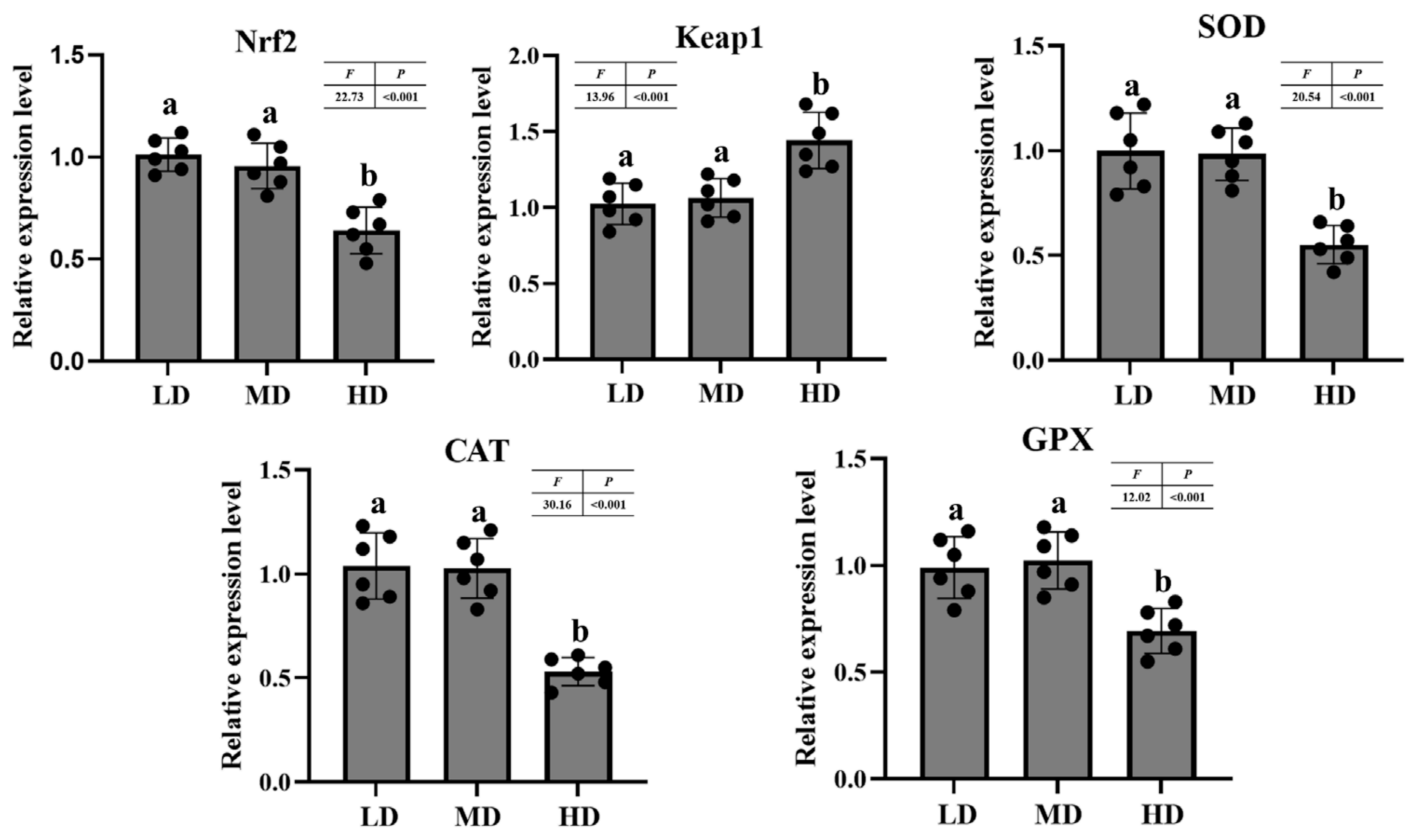
Effects of stocking density on the hepatic expression of antioxidant-related genes in large yellow croaker. The relative mRNA expression levels of Nrf2, Keap1, SOD, CAT, and GPX in the liver were determined by quantitative real-time PCR. Transcript levels were normalized to the reference gene β-actin, and expression in the LD group was set to 1.0. Values are presented as mean ± SE (n = 6). Groups labeled with different letters are significantly different (*p* < 0.05). Dots represent individual biological replicates.

The hepatic expression of antioxidant-related genes was significantly affected by stocking density (Figure 3). The mRNA expression of Nrf2 did not differ significantly between the LD and MD groups (*p* > 0.05), but was significantly decreased in the HD group (*p* < 0.05). In contrast, Keap1 expression was significantly up-regulated in the HD group compared with the LD and MD groups (*p* < 0.05), whereas no significant difference was observed between the LD and MD groups (*p* > 0.05). Consistent with the change in Nrf2 expression, the transcript levels of the antioxidant genes SOD, CAT, and GPX were all significantly lower in the HD group than in the LD and MD groups (*p* < 0.05), with no significant differences between the latter two groups (*p* > 0.05). These results indicate that high stocking density suppressed the hepatic Nrf2-related antioxidant response in large yellow croaker.

### 3.5. Effects of Stocking Density on Hepatic Expression of Inflammation-Related Genes

**Figure 4:**
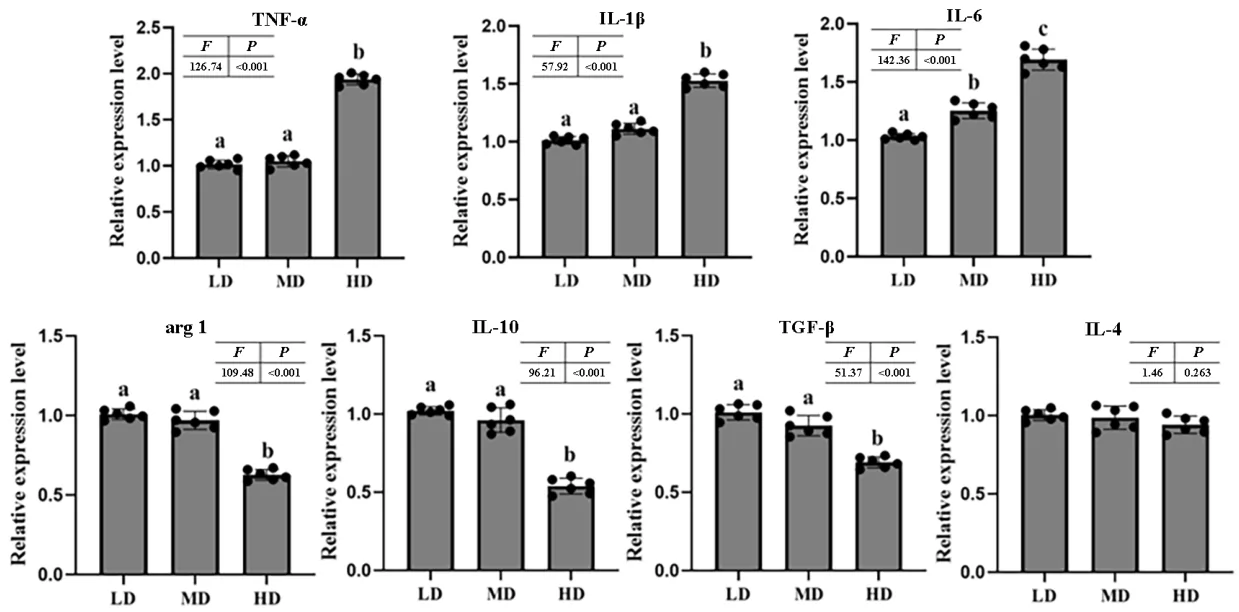
Effects of stocking density on hepatic expression of inflammation-related genes in large yellow croaker. The relative mRNA levels of pro-inflammatory cytokines (TNF-α, IL-1β and IL-6) and anti-inflammatory factors (IL-10, arg1, TGF-β and IL-4) in the liver were determined by quantitative real-time PCR. Transcript levels were normalized to the reference gene β-actin, and the expression in the LD group was set as 1.0. Values are presented as mean ± SE (*n* = 6). Groups labeled with different letters are significantly different (*p* < 0.05). Dots represent individual biological replicates.

The hepatic mRNA expression of pro-inflammatory cytokines, including TNF-α and IL-1β, did not differ significantly between the LD and MD groups (*p* > 0.05) but was significantly up-regulated in the HD group (*p* < 0.05) (Figure 4). In addition, the expression of IL-6 was significantly increased with increasing stocking density (*p* < 0.05), with the HD group showing the highest level and the MD group showing a moderate but significantly higher level than the LD group (*p* < 0.05). In contrast, the expression of the anti-inflammatory factors IL-10, Arg1, and TGF-β was significantly down-regulated in the HD group compared with the LD and MD groups (*p* < 0.05), whereas the expression of IL-4 was not significantly affected by stocking density (*p* > 0.05).

### 3.6. Effects of Stocking Density on Hepatic NF-κB Signaling Pathway

**Figure 5:**
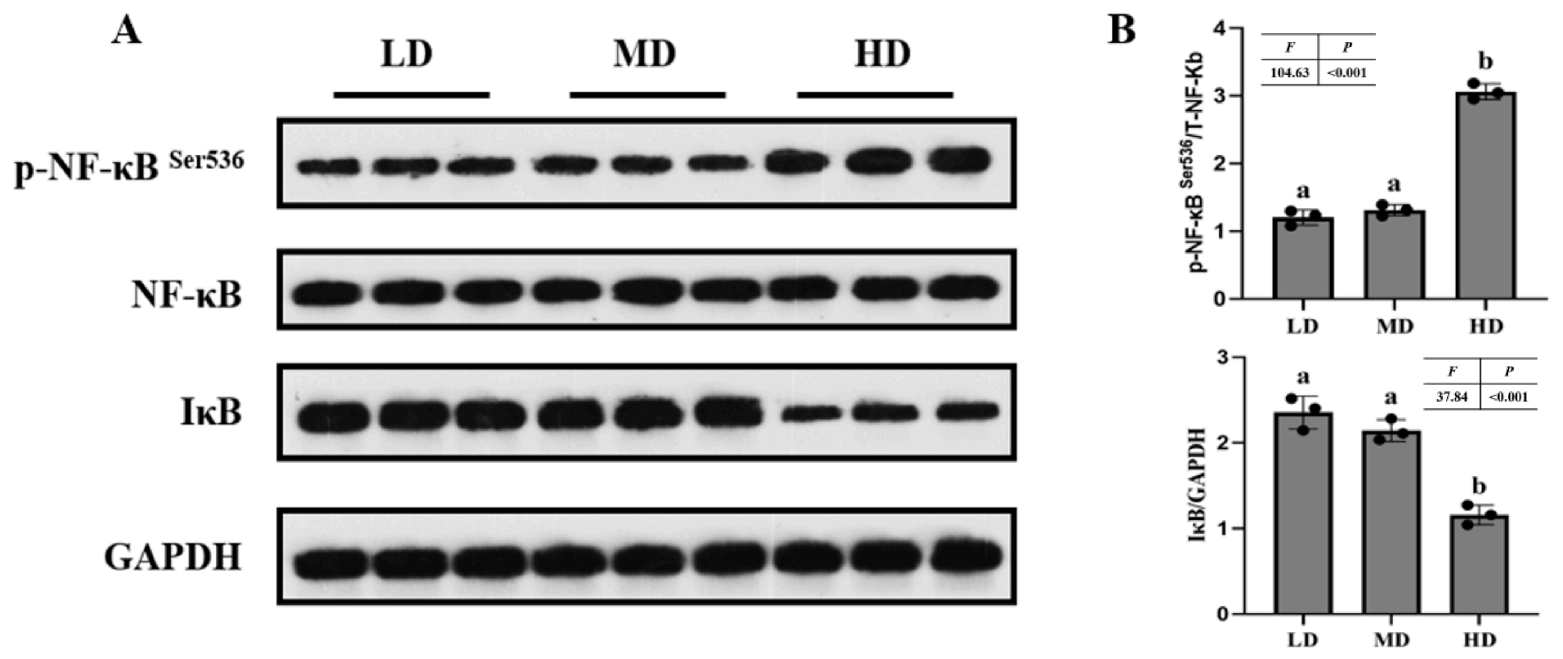
Effects of stocking density on the hepatic NF-κB signaling pathway in large yellow croaker. (**A**) Representative Western blot bands of phosphorylated NF-κB p65 (Ser536), total NF-κB p65, IκB, and GAPDH. (**B**) Quantitative analysis of the p-NF-κB p65/total NF-κB p65 ratio and IκB abundance relative to GAPDH. Values are presented as mean ± SE (*n* = 3), and the LD group was set to 1.0. Groups labeled with different letters are significantly different (*p* < 0.05). Dots represent individual biological replicates.

To further explore the molecular mechanism underlying the density-induced inflammatory response, the activation status of the NF-κB signaling pathway in the liver was determined by Western blot (Figure 5). The ratio of phosphorylated NF-κB p65 (Ser536) to total NF-κB was significantly higher in the HD group than in the LD and MD groups (*p* < 0.05), with no significant difference between the LD and MD groups (*p* > 0.05) (Figure 5B). Conversely, the protein abundance of IκB was significantly decreased in the HD group, while it remained high in the LD and MD groups (*p* < 0.05).

### 3.7. Effects of Stocking Density on Intestinal Morphology

**Figure 6:**
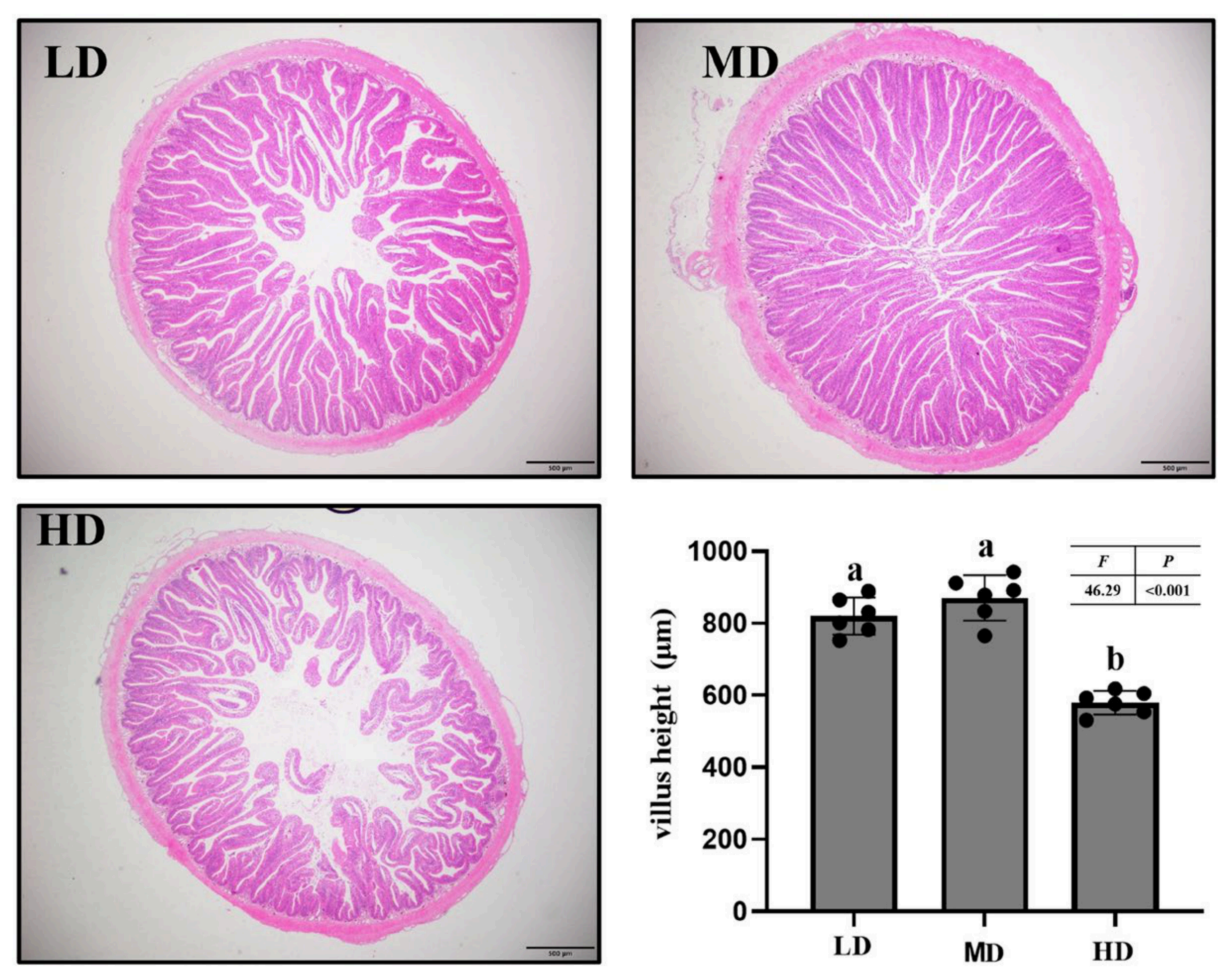
Effects of stocking density on the intestinal morphology of large yellow croaker. The villus height of the mid-intestine was measured on H&E-stained sections. Values are presented as mean ± SE (*n* = 6). Groups labeled with different letters are significantly different (*p* < 0.05). Dots represent individual biological replicates.

The intestinal morphology of large yellow croaker was affected by stocking density (Figure 6). Compared with the HD group, the LD and MD groups exhibited significantly greater intestinal villus height (*p* < 0.05) (Figure 6). The villus height of the MD group tended to be lower than that of the LD group, but the difference was not significant (*p* > 0.05).

### 3.8. Effects of Stocking Density on Intestinal Barrier Function and Permeability

**Figure 7:**
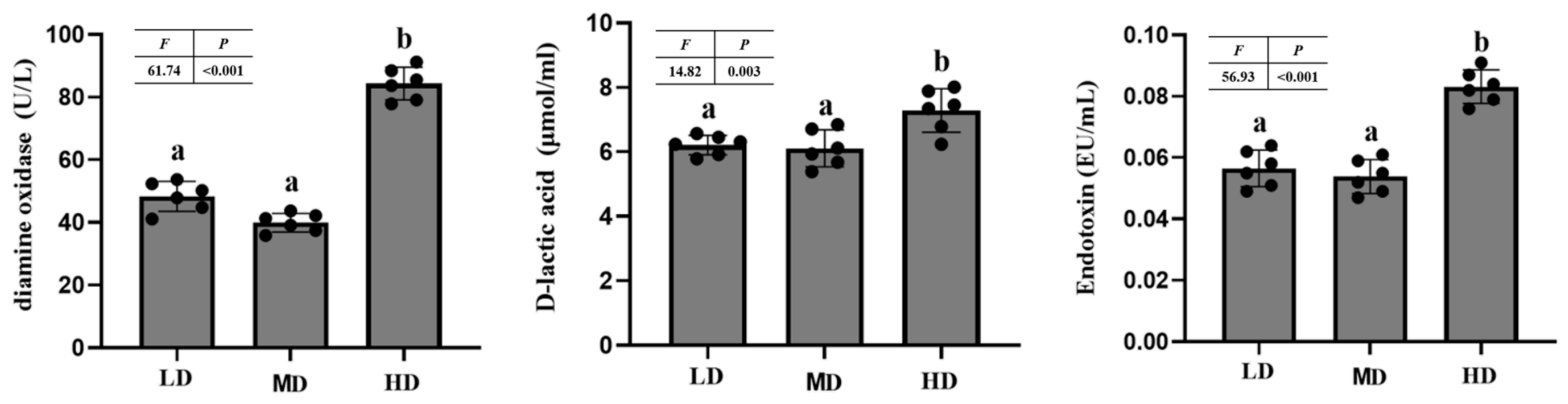
Effects of stocking density on the intestinal barrier function and permeability of large yellow croaker. Serum diamine oxidase (DAO) activity, D-lactate content and endotoxin level were measured after the feeding trial. Values are presented as mean ± SE (*n* = 6). Groups labeled with different letters are significantly different (*p* < 0.05). Dots represent individual biological replicates.

The serum markers of intestinal mucosal integrity and permeability were significantly altered by stocking density (Figure 7). The serum diamine oxidase (DAO) activity did not differ between the LD and MD groups (*p* > 0.05), but increased significantly in the HD group (*p* < 0.05). Similarly, the serum D-lactate content and endotoxin level were comparable between the LD and MD groups (*p* > 0.05), whereas both were significantly elevated in the HD group (*p* < 0.05).

### 3.9. Effects of Stocking Density on Intestinal Tight Junction Proteins

**Figure 8:**
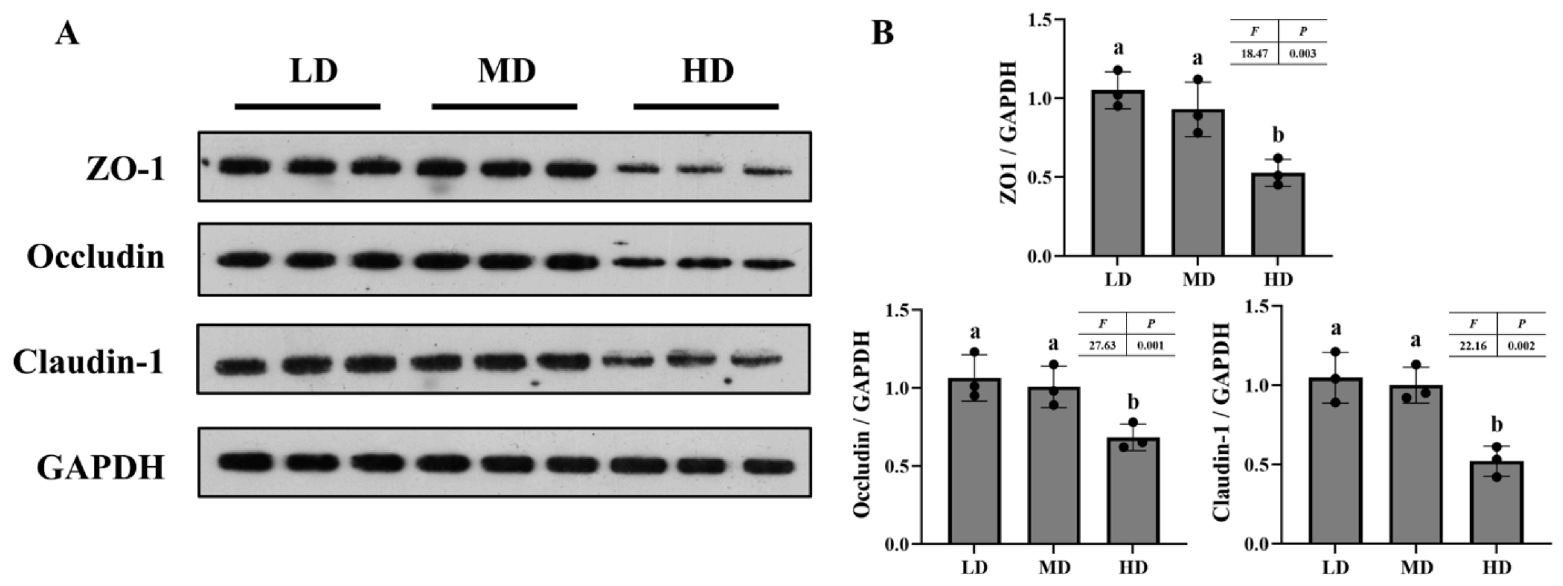
Effects of stocking density on intestinal tight junction proteins in large yellow croaker. (**A**) Representative Western blot bands of ZO-1, Occludin, Claudin-1, and GAPDH. (**B**) Quantitative analysis of ZO-1, Occludin, and Claudin-1 abundance relative to GAPDH. Values are presented as mean ± SE (*n* = 3). Groups labeled with different letters are significantly different (*p* < 0.05). Dots represent individual biological replicates.

To further verify the impairment of the intestinal physical barrier at the protein level, the abundance of tight junction proteins ZO-1, Occludin and Claudin-1 in the intestine was determined by Western blot (Figure 8A). The protein levels of ZO-1, Occludin and Claudin-1 (normalized to GAPDH) did not differ significantly between the LD and MD groups (*p* > 0.05) but were all significantly decreased in the HD group (*p* < 0.05) (Figure 8B).

## 4. Discussion

Stocking density is a double-edged factor in intensive aquaculture: a moderate density maximizes the utilization of rearing space, whereas an excessive density acts as a chronic stressor that compromises fish growth, welfare and health [41,42]. In the present study, high stocking density significantly depressed the growth performance and feed utilization of large yellow croaker, as reflected by the reduced final body weight, weight gain rate, specific growth rate and feeding rate, and the elevated feed conversion ratio, whereas the medium density group achieved the highest final body weight. Similar density-dependent growth depression has been reported in a variety of farmed fish, such as turbot [8] and Chinese sturgeon [9], as well as in large yellow croaker [36]. The reduced feeding rate in the HD group suggests that intensified competition for space and feed, together with chronic stress, suppressed the feeding motivation of fish, thereby reducing nutrient intake. Meanwhile, the increased feed conversion ratio indicates that a greater proportion of the ingested energy was diverted to cope with stress rather than deposited for growth. Notably, the survival rate of the HD group was also significantly reduced, demonstrating that the adverse effects of high stocking density extended beyond growth impairment to threaten fish health. The generally comparable growth performance and most physiological responses of the MD group relative to the LD group suggest that an initial density of 8.64 kg/m^3^ was largely tolerated under the present experimental conditions and allowed more efficient utilization of cage volume. However, the elevated hepatic IL-6 expression in the MD group indicates that some early inflammatory responses may already occur at this density.

Circulating cortisol and glucose are classical primary and secondary indicators of the fish stress response [43]. In the present study, both parameters were significantly elevated in the HD group, indicating that large yellow croaker reared at 11.55 kg/m^3^ experienced chronic crowding stress through sustained activation of the HPI axis [17]. Elevated cortisol promotes gluconeogenesis and glycogenolysis to mobilize glucose as an energy substrate [44], which meets the increased energy demand under stress but concurrently reduces the energy available for somatic growth, providing a physiological explanation for the growth depression observed in the HD group. Consistent elevations of cortisol and glucose under high stocking density have been reported in large yellow croaker and other farmed species [12]. Chronic stress is closely associated with oxidative disturbance because sustained stress responses can increase ROS generation and challenge cellular antioxidant defenses [18]. In the present study, serum T-AOC and SOD activity were significantly decreased, whereas MDA content was increased in the HD group, indicating impaired systemic antioxidant capacity and enhanced lipid peroxidation under high-density conditions. Importantly, these biochemical changes were accompanied by marked alterations in hepatic antioxidant-related gene expression. The mRNA expression of Nrf2 was significantly down-regulated, whereas that of its negative regulator Keap1 was significantly up-regulated in the HD group. In parallel, the expression levels of the antioxidant genes SOD, CAT, and GPX were significantly reduced.

The Keap1/Nrf2 system is a central component of the cellular defense against oxidative stress. Under basal conditions, Keap1 facilitates the ubiquitination and proteasomal degradation of Nrf2, whereas oxidative stimuli can stabilize Nrf2 and promote its nuclear accumulation and antioxidant transcriptional response [20]. The Nrf2/Keap1 system also participates in the regulation of antioxidant defenses in aquatic organisms, including genes associated with SOD, CAT, and GPX-mediated ROS detoxification [22]. Therefore, the simultaneous down-regulation of Nrf2, SOD, CAT, and GPX and up-regulation of Keap1 observed in the HD group suggest an impaired hepatic Nrf2-related antioxidant response under prolonged crowding stress. This interpretation is further supported by the corresponding decrease in serum T-AOC and SOD activity and increase in MDA, providing complementary transcriptional and biochemical evidence of disrupted redox homeostasis.

Previous studies have shown that antioxidant responses to environmental stress in fish can vary with stress intensity, duration, tissue, and physiological condition. In large yellow croaker, environmental stressors have been reported to alter the expression of Nrf2, Keap1, SOD, CAT, and GPX as well as antioxidant enzyme activities [45]. The differences between these studies and the present results may reflect differences in stress type and duration. In the present 8-week experiment, the coordinated reduction in antioxidant-related gene expression and systemic antioxidant capacity in the HD group suggests that prolonged high-density exposure compromised the capacity of the antioxidant defense system to maintain redox homeostasis. Nevertheless, because Nrf2 activity is strongly regulated at the protein level, particularly through protein stabilization and nuclear translocation, the present transcriptional data should be interpreted as evidence of an altered Nrf2-related antioxidant response rather than direct demonstration of Nrf2 pathway inhibition [20].

Chronic stress-induced immunosuppression is frequently accompanied by persistent low-grade inflammation, and the balance between pro- and anti-inflammatory cytokines determines the outcome of the inflammatory response [25]. In the present study, the hepatic expression of the pro-inflammatory cytokines TNF-α and IL-1β was significantly up-regulated only in the HD group, whereas IL-6 expression increased in a dose-dependent manner, with the MD group already showing a significantly higher level than the LD group. This divergence suggests that IL-6 is more sensitive to crowding stress than TNF-α and IL-1β in large yellow croaker and may serve as an early molecular indicator of density-induced inflammation. Conversely, the anti-inflammatory factors IL-10, Arg1 and TGF-β were significantly down-regulated in the HD group, while IL-4 remained unaffected, indicating that anti-inflammatory factors differ in their sensitivity to stocking density. A similar pattern of enhanced pro-inflammatory and suppressed anti-inflammatory cytokine expression has been reported in grass carp subjected to long-term crowding stress [10]. Mechanistically, the present Western blot results demonstrated that high stocking density significantly increased the ratio of phosphorylated NF-κB to total NF-κB and decreased the IκB protein abundance in the liver. Since IκBα degradation results in the nuclear translocation of NF-κB dimers, which drive the transcription of pro-inflammatory cytokine genes [46], the concurrent IκB reduction, p65 phosphorylation and cytokine up-regulation strongly indicate that the NF-κB signaling pathway mediates the hepatic inflammatory response of large yellow croaker induced by high stocking density. Persistent NF-κB activation coupled with the withdrawal of anti-inflammatory signals would shift the hepatic immune milieu toward a chronic inflammatory state, which may contribute to the reduced survival observed in the HD group.

The intestine is a primary target of husbandry stressors, and its structural and barrier integrity is essential for nutrient absorption and health maintenance in fish [29,47]. In the present study, high stocking density significantly shortened the intestinal villus height of large yellow croaker, indicating a reduced absorptive surface area that may partially account for the impaired feed utilization and growth. More importantly, the present study provides, to our knowledge, the first evidence that high stocking density disrupts the intestinal physical barrier of large yellow croaker at the protein level: the abundance of the tight junction proteins ZO-1, Occludin and Claudin-1 was significantly decreased in the HD group. Tight junction complexes seal the paracellular space between intestinal epithelial cells, and their down-regulation directly compromises the selective permeability of the mucosa. Consistently, serum DAO activity and D-lactate and endotoxin levels, which are sensitive circulating markers of intestinal mucosal injury and permeability, were all significantly elevated in the HD group. The parallel changes in villus morphology, tight junction proteins, and serum permeability markers provide convergent evidence of impaired intestinal barrier integrity and increased intestinal permeability in large yellow croaker exposed to high stocking density. Similar density-induced intestinal damage has been described in gilthead sea bream [13] and largemouth bass [48], although tight junction proteins were not examined in those studies.

The concurrent disruption of intestinal barrier integrity and activation of hepatic NF-κB signaling in the HD group raises the possibility of communication along the gut–liver axis. Increased intestinal permeability may facilitate the entry of gut-derived endotoxin into the circulation, which could subsequently contribute to hepatic inflammatory signaling [49,50]. It is therefore plausible that a vicious cycle operates under high stocking density. Crowding stress and oxidative damage first injure the intestinal mucosa and down-regulate tight junction proteins; the resulting endotoxemia then activates hepatic NF-κB-mediated inflammation; and the ensuing systemic inflammatory response, together with stress-related metabolic alterations, further aggravates intestinal injury. This hypothesis is supported by the parallel elevation of serum endotoxin and hepatic NF-κB activation in the HD group and provides a mechanistic framework linking intestinal barrier dysfunction to systemic inflammation under crowding conditions.

Under the present experimental conditions, an initial stocking density of 8.64 kg/m^3^ did not produce the marked adverse responses observed at 11.55 kg/m^3^. This observation is generally consistent with the density range suggested by Yu et al. [36] for large yellow croaker. It should be noted that several limitations of the present study should be acknowledged. First, only one fish size and culture condition were investigated. Given that the optimal stocking density may vary with fish size, season, and culture system, caution should be exercised when extrapolating the present findings to other farming conditions. Second, mortality in the HD group reduced the actual density during the experiment, which may have partially alleviated crowding stress and consequently underestimated the adverse effects of sustained high stocking density. Finally, the intestinal microbiota, which may contribute to intestinal barrier disruption, was not investigated.

## 5. Conclusions

In conclusion, high stocking density impaired growth and survival and induced pronounced physiological stress and oxidative imbalance in cage-cultured large yellow croaker. These changes were accompanied by up-regulation of hepatic Keap1 and down-regulation of Nrf2 and the antioxidant genes SOD, CAT, and GPX, suggesting suppression of the Nrf2-related antioxidant response under prolonged crowding stress. High stocking density was also associated with activation of hepatic NF-κB inflammatory signaling and impairment of intestinal barrier integrity, whereas fish reared at medium density showed generally comparable responses to those reared at low density for most measured endpoints. Collectively, these findings indicate that disruption of redox homeostasis, hepatic inflammatory signaling, and intestinal barrier function are important physiological responses associated with crowding-induced impairment in large yellow croaker. Further studies incorporating additional density gradients, different fish sizes and environmental conditions, as well as analyses of the intestinal microbiota and gut–liver signaling, are warranted to refine stocking-density management strategies.

## Figures and Tables

**Table 1:** Proximate composition of the experimental diet (% dry matter).

Composition	Crude Protein	Crude Fat	Crude Fiber	Crude Ash	Phosphorus	Lysine	Moisture
Content (%)	45	6	5	15	2.3	2.6	10

**Table 2:** Primer sequences used for quantitative RT-PCR.

Gene	Primers (5′-3′)	Source or GenBank Accession Number
TNF-α	F: ACACCTCTCAGCCACAGGAT	XM_010745990
	R: CCGTGTCCCACTCCATAGTT	
IL-1β	F: CAGCTGTTCTCAAGTATGTGGC	XM_010736551.3
	R: GTTGTAAATAGTGGGTGTGTCG	
IL-6	F: TGAAGGCTCCGACGAAATG	KU140675
	R: GTCCAGTAGGCTAAACTGCTATC	
IL-10	F: AGTCGGTTACTTTCTGTGGTG	XM_010738826.3
	R: TGTATGACGCAATATGGTCTG	
Arg1	F: AACCACCCGCAGGATTACG	Cen et al. [40]
	R: AAACTCACTGGCATCACCTCA	
TGF-β	F: AGCAACCACCGTACATCCTG	XM_027280465.1
	R: AGGTATCCCGTTGGCTTGTG	
IL-4	F: TGGTACTGCTGGTCAATCCG	KU885454
	R: TTTTGCCTTCAGCCAGATGT	
Nrf2	F: GATGGAAATGGAGGTGATGC	XM_010737768.3
	R: CATGTTCTTTCTGTCGGTGG	
Keap1	F: CGGGGAGTCTCACAGCATT	XM_019274257.2
	R: CTTCCAACATAATCCAAACACC	
SOD	F: ATGGTGATCCACGAGAAGGC	NM_001303360.1
	R: CTACCAGCGTTGCCAGTCTT	
CAT	F: GACGCTCTACTGTTCCCGTC	XM_010735178.3
	R: GCAAACCTCGATCGCTGAAC	
GPX	F: GACTCGTTATTCTGGGTGTTCCCTGTA	KY689026.1
	R: CCATTCCCTGGACGGACATACTTC	
β-Actin	F: CTACGAGGGTTATGCCCTGCC	GU584189.1
	R: TGAAGGAGTAACCGCGCTCTGT	

Note: TNF-α, tumor necrosis factor-α; IL-1β, interleukin-1β; IL-6, interleukin-6; IL-10, interleukin-10; Arg1, arginase 1; TGF-β, transforming growth factor-β; IL-4, interleukin-4; Nrf2, nuclear factor erythroid 2-related factor 2; Keap1, Kelch-like ECH-associated protein 1; SOD, superoxide dismutase; CAT, catalase; GPX, glutathione peroxidase; F, forward primer; R, reverse primer.

**Table 3:** Effects of stocking density on growth performance and feed utilization of large yellow croaker.

Item	LD	MD	HD	*F*	*p*
Initial fish number per cage	90	135	180	/	/
Initial density (kg/m^3^)	5.73	8.64	11.55	/	/
Final density (kg/m^3^)	7.43	11.50	12.65	/	/
Initial body weight (g)	286.27 ± 1.93	287.98 ± 1.57	288.64 ± 2.12	1.26	0.349
Final body weight (g)	412.91 ± 2.56 ^b^	424.23 ± 2.26 ^a^	395.30 ± 2.21 ^c^	115.61	<0.001
Weight gain rate (%)	44.24 ± 1.57 ^a^	47.31 ± 1.08 ^a^	36.95 ± 1.25 ^b^	49.07	<0.001
Specific growth rate (%/day)	0.65 ± 0.02 ^a^	0.69 ± 0.01 ^a^	0.56 ± 0.02 ^b^	44.33	<0.001
Feeding rate (%/day)	1.06 ± 0.04 ^a^	1.07 ± 0.02 ^a^	0.94 ± 0.05 ^b^	10.47	0.011
Feed conversion ratio	1.15 ± 0.07 ^a^	1.16 ± 0.06 ^a^	1.29 ± 0.04 ^b^	5.44	0.045
Survival rate (%)	89.92 ± 2.07 ^a^	90.23 ± 1.79 ^a^	80.16 ± 1.98 ^b^	25.87	0.001

Values are means ± SE of three replicate cages (*n* = 3). Values in the same row with different superscript letters are significantly different (*p* < 0.05). LD, low density; MD, medium density; HD, high density.

## Data Availability

Data will be made available on request.
